# UPLC–MS Triglyceride Profiling in Sunflower and Rapeseed Seeds

**DOI:** 10.3390/biom9010009

**Published:** 2018-12-27

**Authors:** Alina Chernova, Rim Gubaev, Pavel Mazin, Svetlana Goryunova, Yakov Demurin, Lyudmila Gorlova, Anna Vanushkina, Waltraud Mair, Nikolai Anikanov, Elena Martynova, Denis Goryunov, Sergei Garkusha, Zhanna Mukhina, Philipp Khaytovich

**Affiliations:** 1 Center of Life Sciences, Skolkovo Institute of Science and Technology, Nobel st. 3, Building 1, Moscow 121205, Russia; rimgubaev@gmail.com (R.G.); iaa.aka@gmail.com (P.M.); svetlana.v.goryunova@gmail.com (S.G.); A.Vanyushkina@skoltech.ru (A.V.); W.Mair@skoltech.ru (W.M.); N.Anikanov@skoltech.ru (N.A.); E.Martynova@skoltech.ru (E.M.); D.Goryunov@skoltech.ru (D.G.); P.Khaitovich@skoltech.ru (P.K.); 2 Institute for Information Transmission Problems (Kharkevich Institute), Russian Academy of Sciences, Moscow 127051, Russia; 3 Faculty of Computer Science, National Research University Higher School of Economics, Moscow 119991, Russia; 4 Institute of General Genetics, Russian Academy of Sciences, Gubkin st. 3, Moscow 119991, Russia; 5 Pustovoit All-Russia Research Institute of Oil Crops, Filatova st. 17, Krasnodar 350038, Russia; yakdemurin@yandex.ru (Y.D.); lagorlova26@yandex.ru (L.G.); 6 Belozersky Institute of Physico-Chemical Biology, Moscow State University, Leninskie Gory 1, Building 40, Moscow 119234, Russia; 7 All-Russia Rice Research Institute, Belozerny 3, Krasnodar 350921, Russia; arrri_kub@mail.ru (S.G.); agroplazma@gmail.com (Z.M.)

**Keywords:** ultraperformance liquid chromatography–mass spectrometry (UPLC–MS), triglycerides, sunflower, rapeseed, winter-type rapeseed, spring-type rapeseed, cold resistance, freezing tolerance

## Abstract

Sunflower and rapeseed are among the most important sources of vegetable oil for food and industry. The main components of vegetable oil are triglycerides (TAGs) (about 97%). Ultra- performance liquid chromatography coupled with mass spectrometry (UPLC–MS) profiling of TAGs in sunflower and rapeseed has been performed and the TAG profiles obtained for these species have been compared. It has been identified that 34 TAGs are shared by sunflower and rapeseed. It was demonstrated that TAGs 52:2, 52:5, 52:6, 54:3; 54:4, 54:7, 56:3, 56:4, and 56:5 had the highest variability levels between sunflower and rapeseed with the higher presence in rapeseed. TAGs 50:2, 52:3, 52:4, 54:5, and 54:6 also showed high variability, but were the most abundant in sunflower. Moreover, the differences in TAG composition between the winter-type and spring-type rapeseed have been revealed, which may be associated with freezing tolerance. It was shown that winter-type rapeseed seeds contain TAGs with a lower degree of saturation, while in spring-type rapeseed highly saturated lipids are the most abundant. These findings may give new insights into the cold resistance mechanisms in plants the understanding of which is especially important in terms of global climate changes.

## 1. Introduction

Vegetable oil has been used by humans for thousands of years [1]. Nowadays, vegetable oils are still widely utilized by the food and chemical industry as well as acting an important source for fuel production [2,3]. Plants generally accumulate oil in their seeds and fruits to provide energy for germination and the early stages of seedling development. Triacylglycerols or triglycerides (TAGs) are the main component (up to 97%) of vegetable oil and play various physiological roles in plants including energy balance maintenance, lipid homeostasis, and growth and development [4,5]. Triglycerides are composed of three fatty acids (FAs) esterified to glycerol, where FAs determine the biochemical properties of TAGs [6]. Fatty acids differ by the carbon chain length and saturation level. The number of double bonds and their position determine oil heat stability and its nutritional properties, while FA chain length affects the detergent properties of the soap derived from the oil which can make vegetable oil so advantageous for special industrial needs [7].

Rapeseed and sunflower take the positions three and four in the global production of vegetable oil after oil palm and soybean [8]. Throughout the sunflower and rapeseed crop improvement history, seed oil content and oil quality were the traits which have drawn most attention. Triglycerides composition is one of the main directions in sunflower and rapeseed selection [9,10,11]. Quality of sunflower oil is usually determined by the ratio between oleic and linoleic acids. The typical fatty acid components of sunflower oil are linoleic acid (55–65%), oleic acid (20–30%), and the remaining FAs, first of all, palmitic and stearic, and the minor ones [12]. In rapeseed oil, oleic acid usually predominates (about 61%), linoleic acid is the second most abundant (20%), and saturated fatty acids such as palmitic and stearic constitute less than 10%. Since rapeseed oil is widely used in both the food industry and for non-food purposes, rapeseed breeding lines with the varying fatty acid composition are of great value to the oil crop market. One of the most important goals of rapeseed breeding programs is the creation of breeding lines and varieties from which oil with low linolenic acid content (C18:3 ≤ 3%) can be extracted. Low content of polyunsaturated linolenic acid prevents oxidation and rancidification of the oil, which renders possible its use for deep frying. In addition, oil with low linolenic acid content serves as an important source of raw material for biofuel production because of its high stability [13].

In the case of sunflower oil, high oleic acid and low linoleic acid content is also a highly valuable property. Such oil is characterized by a higher degree of oxidative stability, which makes it more suitable for frying, refining, and storage [14,15]. A new promising sunflower oil type is sunflower oil with high oleic content coupled with high stearic acid content. The oil of such type is very stable to oxidation and can serve as a healthy alternative to the palm oil due to its specific properties [16]. 

Since TAG composition determines oil quality as well as many important oil characteristics, its analysis in oilseed crops is of special interest. Previous works in which TAG composition of seeds in different sunflower lines have been studied [17,18]. The knowledge on the content and composition of TAGs in the oil is essential for improving its TAG composition, optimization of the physicochemical properties, and enhancement of the nutritional value of edible oil as well as its industrial properties [19].

Rapeseed varieties can be divided into two types based on the specific features of their growth and development, namely, annual (spring)-type varieties, which are seeded in the spring and complete their life cycle in a single growing season and biennial (winter)-type varieties, which are usually seeded in the autumn, pass the winter as seedlings, and then complete their development the following spring [20]. Winter-type rapeseed has apparently made special adaptations to become cold resistant and freezing tolerant [21]. Previous studies have shown that cold resistance is closely associated with plant cell membranes [22]. Lipid molecules and proteins integrated into the membrane regulate membrane stability. Since membrane lipids and TAGs are produced by the same biosynthetic pathway [7], the knowledge on which storage lipids are associated with cold tolerance in seeds can shed light on the evolution of this trait in plants and its regulating mechanisms.

The development of high-throughput techniques allowed performing massive analysis of oil composition in plants worldwide [4].

In the present study, we applied ultra-performance liquid chromatography–mass spectrometry (UPLC–MS) to TAG profiling in sunflower and rapeseed. This is the first attempt to use a high-throughput technique to explore a Russian oilseed crop germplasm collection. Triglycerides composition in 50 sunflowers and 48 rapeseed lines from the VNIIMK (Pustovoit All-Russian Research Institute of Oil Crops) collection has been compared and the main differences in the TAG content between the breeding lines of these two plants have been revealed. In addition, TAG content and fatty acids saturation levels in the detected TAGs have been compared in the winter-type and spring-type rapeseed. The observed differences may give new insights into the mechanisms of *Brassica napus* cold resistance and freezing tolerance.

## 2. Materials and Methods

### 2.1. Plant Material

Seeds from 50 sunflower (*Helianthus annuus*) and 48 rapeseed (*Brassica napus*) lines (Supplementary Table S1) from the Pustovoit All-Russia Research Institute of Oil Crops Collection (Krasnodar, Russia) were used in the study.

### 2.2. Reagents

Methanol LC-MS (Scharlau, Barcelona, Spain), methyl *tert*-butyl ether HPLC grade (Scharlau, Barcelona, Spain), water UPLC–MS grade (Scharlau, Barcelona, Spain), lipid standards (LPC #791643 and 15:0-18:1-d7 DG #791647; Avanti (Alabaster, AL, USA), acetonitrile LC/MS grade (Fisher Chemical, Loughborough, UK, isopropanol liquid chromatography (LC)–MS grade (Honeywell Fluka, Morris Plains, NJ, USA), ammonium acetate (Honeywell Fluka, Morris Plains, NJ, USA), formic acid 98–100% LC–MS grade (LiChropur Merck Millipore, Burlington, MA, USA), and zirconium oxide beads (Bertin Instruments, Montigny-le-Bretonneux, France) were used to perform the study.

### 2.3. UPLC–MS Analysis

For lipid extraction, 10 mg (for each line) of *sunflower* (1 sample—1 seed) and *rapeseed* (1 sample—several seeds) seeds were homogenized using six 2.8 mm zirconium oxide beads in the Precellys Evolution homogenizer (Bertin Instruments, Montigny-le-Bretonneux, France) coupled with the Cryolys cooling system filled with dry ice at the temperature not exceeding 10 °C. Homogenization parameters were as follows: 6800 rpm, three times for 20 s, pause 30 s. 400 µL of methanol/methyl *tert*-butyl ether mixture (1:3, *v*:*v*) was added to each sample prior to homogenization. After homogenization, another 400 µL of methanol/methyl *tert*-butyl ether mixture was added, and samples were vortexed. After sonication for 10 min in the ice-cooled ultrasonic bath and incubation at 4 °C for 30 min with shaking, samples were transferred into new 1.5 mL Eppendorf tubes (Eppendorf, Hamburg, Germany), and 560 mL of water/methanol mixture (3:1, *v*:*v*) was added. After the addition of water/methanol, samples were vortexed for 10 min and centrifuged at 12,700 rpm at 4 °C for 10 min. This led to the separation of two phases: the lipophilic phase and the polar phase. The upper lipophilic phase was collected and vacuum-dried in Concentrator plus (Eppendorf, Hamburg, Germany) at 30 °C for 1.5 h. The pellet was stored at −80 °C prior to analysis. This protocol was based on the protocol described by Giavalisco et al [23].

To prepare samples for the injection into the UPLC–MS system, the dried extracts were first resuspended in 200 μL of acetonitrile/isopropanol mixture (70:30, *v*:*v*), vortexed for 10 s, and then incubated for 10 min at 4 °C with shaking, followed by sonication for 10 min on ice, and centrifugation at 12,700 rpm for 7 min at 4 °C. After the completion of these procedures, the final dilution with acetonitrile/isopropanol (70:30, *v*:*v*) was prepared in MS vials (a 25-fold dilution was used).

Samples were analyzed by mass-spectrometry coupled with reversed phase ultra-performance liquid chromatography (ACQUITY UPLC System; Waters, Milford, MA, USA) in the positive ionization mode on Q-TOF Maxis Impact II mass-spectrometer (Bruker Daltonik, Bremen, Germany) (UPLC–MS). The settings were as follows: ion polarity, positive; scan mode, MS; mass range, 50–1200 *m*/*z*, and spectra rate, 2 Hz.

Ultra-performance liquid chromatography separation was carried out on the C8 Acquity Beh column (2.1 mm × 100 mm, 1.7 µm particle size; Waters) and the Acquity BEH C8 1.7 μm Vanguard precolumn (Waters) at 60 °C. To prepare UPLC gradient, mobile phases consisted of two solvents: solvent A: 1% of 1 M NH_4_Ac solution and 0.1% formic acid in water, and solvent B: acetonitrile/isopropanol (7:3, *v*:*v*; 1% of 1 M NH_4_Ac solution; and 0.1% formic acid), with the injection volume of 3 µL. The following gradient profile was applied: 55% B, 1 min; linear gradient from 55% B to 80% B, 3 min; linear gradient from 80% B to 85% B, 8 min; linear gradient from 85% B to 100% B, 3 min. After washing the column for 4 min 50 s with 100% B, the proportion of buffer B in the mixture was set back to 55%, and the column was re-equilibrated for 4 min 10 s (24.5 min total run time), with the mobile phase flow rate of 400 µL/min.

Control samples were analyzed in the non-targeted MS2 scan mode.

### 2.4. Data Processing and Analysis

Xcms package [24] was used for UPLC–MS data processing. Optimal parameters were chosen with the aid of the IPO (Isotopologue Parameter Optimization) package [25]. Peak intensities were obtained using the peak Table function implemented in the xcms package with method = ‘maxint’. As a result, a set of peaks characterized by the retention time, mass divided by charge, and intensity were obtained for each sample.

**Figure 1:**
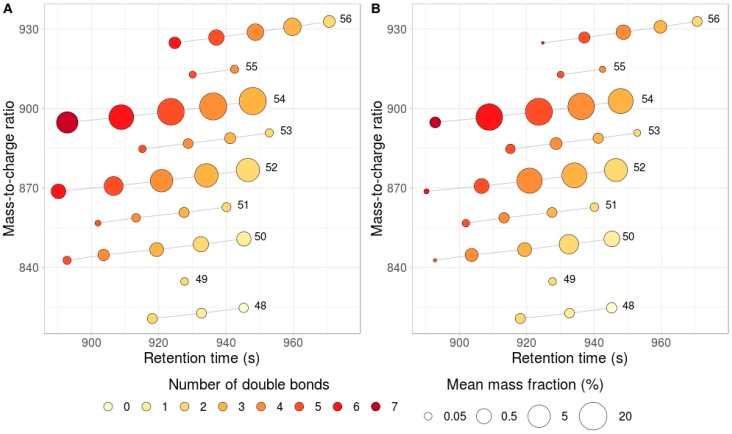
Retention time –mass to charge plot for (**A**) rapeseed and (**B**) sunflower. Single dot corresponds to a single triglyceride, retention time (seconds) and *m*/*z* are shown on *x*- and *y*-axis, respectively. Color scale represents the number of double bonds. Circle size represents the mass fraction. Grey lines connect TAGs with the same fatty acids carbon chain length (indicated at the right end of each line).

To identify peaks that correspond to triacylglycerols, all possible chemical formulas that correspond to TAGs with the total chain length varying between 30 and 85 carbon atoms (n) and with the number of double bonds varying from 0 to 12 (k) defined as C_n+3_H_2(n−k+1)_O_6_ were generated. Isomers were not distinguished on this step and were annotated as a single TAG species. For example, 54:6 denotes a TAG with the total length of fatty acid chains and with the total number of double bonds equal to 54 and 6, respectively. MS2 spectra were used to identify the exact fatty acid content of each TAG. The masses for all possible TAGs with the ammonia adduct were calculated, and all peaks that matched these masses within 20 ppm (for two masses m1 and m2 ppm = abs(m1 − m2)/max(m1, m2) × 10^6^) were found. Then peaks were manually selected based on the net-like patterns as described by Giavalisco et al. [23]. Briefly, it has been observed that the extension of the total chain length increases retention time, while the addition of a double bond reduces retention time. As a result, TAG peaks form a net-like pattern on RT-m/z plots (Figure 1). 

To remove contaminating TAGs, all peaks with the ratio of mean intensity in plant samples to mean intensity in blank samples below four were removed (Supplementary Figure S1). To obtain TAG mass fractions, the intensity of each TAG (Table S2) was divided by the sum of intensities for all TAGs in the given sample. Wilcoxon test with the Benjamini–Hochberg (BH) correction was used to assess the statistical significance of the observed differences.

Statistical analysis and data visualization were carried out using R [26].

## 3. Results and Discussion

Triglycerides in 50 sunflowers and 48 rapeseed lines (Supplementary Table S1) were measured by UPLC–MS. After the obtained measurement results were filtered, 34 TAGs in common between sunflower and rapeseed were identified (Supplementary Table S3A,B). The results are presented in Figure 1A,B. The total amount of double bonds per TAG varied from 1 to 7. All TAGs showed variability in their intensity. The most intense TAGs in sunflower were 54:3, 54:4, 54:5, 54:6, 54:7, 52:2, 52:3, and 52:4 and in rapeseed, 54:3, 54:4, 54:5, and 54:6. Based on their *m*/*z* and retention times, all TAGs may be represented as a net-like pattern. The net-like pattern obtained in this work is in good correspondence with the results obtained previously by Hummel et al. [27]. It can be seen in Figure 1 that TAGs with the same FA chain length lie on the same line, and all identified TAGs were taken together to form parallel lines on the graph. From bottom to top these lines contain lipids with the increasing fatty acid chain length, while the number of double bounds decreases from left to right.

It can be seen, therefore, that TAG patterns may serve as the fingerprints of oil which can be obtained from the analyzed samples. This was also suggested previously by Fernández-Moya et al. [17].

### 3.1. Sunflower versus Rapeseed

**Figure 2:**
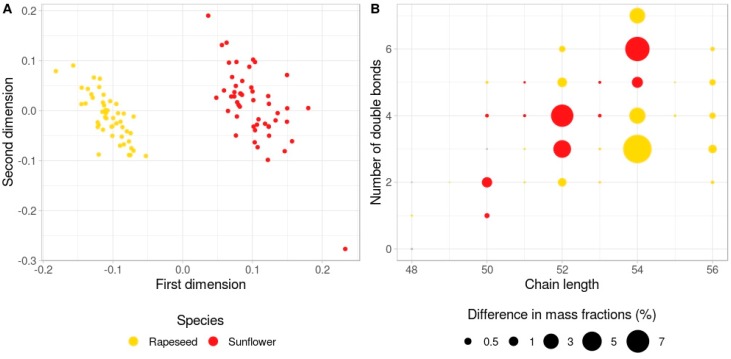
Triglyceride comparison between sunflower and rapeseed. (**A**) Multidimension scaling plot (one minus Spearman correlation coefficient was used as the distance, two dimensions) of samples demonstrating a clear segregation of rapeseed and sunflower samples. (**B**) The difference in the TAG mass fractions between rapeseed and sunflower. Color indicates which species possess a higher amount of a certain TAG. Circle size represents the absolute difference in mass fractions. Grey circles correspond to the TAGs that do not show statistically significant differences.

The intensities of TAGs in sunflower were compared with those in rapeseed. Multidimensional scaling (MDS) analysis revealed clear clustering based on all 34 TAGs common for the two plants (Figure 2A). Among them, 31 TAGs exhibited significant differences between sunflower and rapeseed (Supplementary Table S3A, Figure 2B). Triglycerides 52:2, 52:5, 52:6, 54:3; 54:4, 54:7, 56:3, 56:4, and 56:5 showed the highest variability levels between sunflower and rapeseed with the higher presence in rapeseed. Triglycerides 50:2, 52:3, 52:4, 54:5, and 54:6 also showed substantially high variability, but were the most abundant in sunflower.

**Figure 3:**
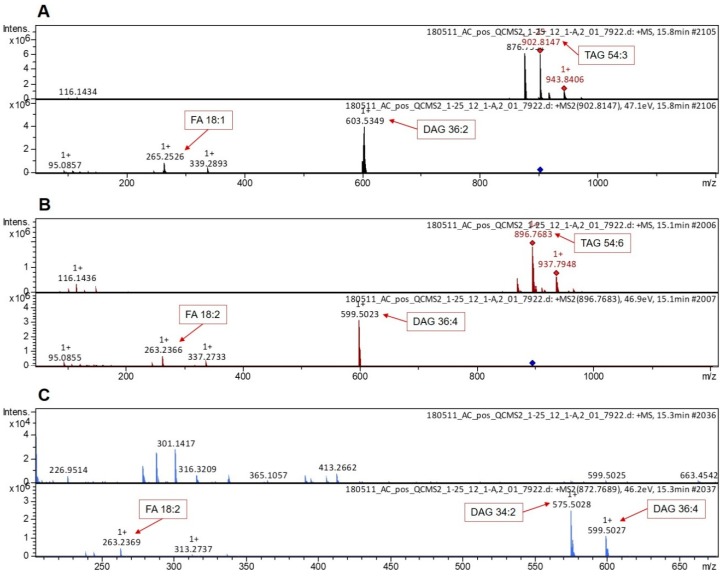
MS2 fragmentation spectra with fragment annotation. (**A**) Fragmentation spectrum for TAG 54:3. (**B**) Fragmentation spectrum for TAG 54:6. (**C**) Fragmentation spectrum for TAG 52:4. DAG: Diglycerides.

Control samples were analyzed in the MS2 scan mode in order to reveal the precise FA content in TAGs. This allowed to carry out a more detailed comparison between the sunflower and rapeseed TAGs. Triglycerides 54:3 is the most abundant in rapeseed. According to the MS2 results, this TAG contains three FAs 18:1 (oleic acid) (fragmentation pattern is depicted in Figure 3A), which is in good correspondence with the literature data [13]. The most abundant in sunflower are TAGs 52:4 and 54:6, which according to the MS2 data, contain two linoleic (18:2) and one palmitic (16:0) acids and three linoleic acids, respectively (fragmentation pattern is depicted in Figure 3B,C). Linoleic acid is the most abundant fatty acid in sunflower on the whole.

### 3.2. Spring-Type versus Winter-Type Rapeseed

**Figure 4:**
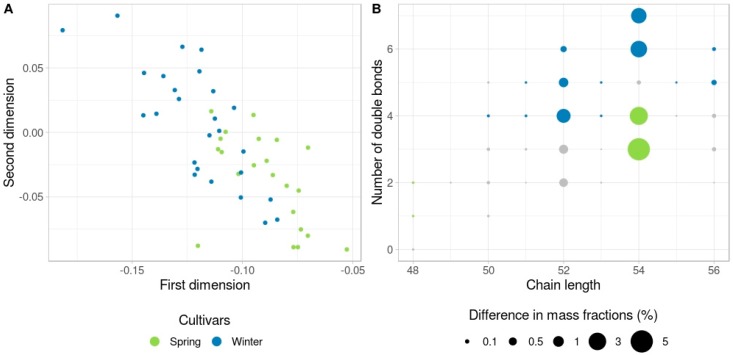
TAG comparison between winter and spring rapeseed. (**A**) Multidimensional scaling (MDS) plot of rapeseed samples (one minus Spearman correlation coefficient was used as the distance, two dimensions) demonstrating a clear segregation of winter and spring rapeseed samples. (**B**) The difference in the TAG mass fractions between winter and spring rapeseed. Color indicates which cultivar possesses a higher amount of certain TAG. Circle size represents the absolute difference in mass fractions. Grey circles correspond to the TAGs that do not show statistically significant differences.

Triglycerides intensities in spring-type and winter-type rapeseed lines were compared. Multidimensional scaling analysis revealed differences between these two groups (Figure 4A) with 17 TAGs demonstrating significant variability between the spring and winter lines (Supplementary Table S3B, Figure 4B). Interestingly, the TAGs with higher amount of double bonds (52:4, 52:5, 52:6, 54:6, and 54:7) are more abundant in the seeds of winter lines, while the TAGs with higher level of FA chain saturation (48:1, 48:2, 54:2, and 54:3) are more abundant in spring lines. These results may most likely be accounted for by the chemical properties of FAs. The degree of saturation is highly important for the FA crystallization processes. Triglycerides containing double bonds have significantly lower melting points than completely saturated TAGs [28].

Winter-type rapeseed seeds were shown to contain TAGs with a lower degree of saturation, which probably has an impact on winter-type rapeseed cold resistance. Freezing tolerance is one of the most important plant traits allowing them to survive in the low temperatures. Freezing tolerance correlates well with winter survival [29]. The unsaturated fatty acid content of the plasma membrane is associated with cold resistance in plants. Plants with higher content of unsaturated fatty acids in their membranes are more resistant to cold [30]. Storage lipids, TAGs, are produced by the extension of the membrane-lipid biosynthetic pathway that is why in the majority of plants, TAGs found in most seeds usually contain the same acyl groups as those found in membrane lipids. At the same time, while membrane lipid composition is highly conservative across plant species, the variability in the fatty acyl chains found in the seed oil is very high [7]. There exist a broad range of factors affecting freezing tolerance in *Brassica* species with all classes of macromolecules being involved, lipids among them [21]. It may appear that the FA content patterns detected in the seeds of winter-type and spring-type rapeseed are just a footprint of the total membrane lipid content specific for the winter-type and spring-type plants, since during the germination period in oilseed plants storage lipids, mainly TAGs, are catabolized with polar lipids, phospholipids, and galactolipids being synthesized de novo [18]. But it cannot be also excluded that TAGs play their own specific role in the seeds of winter-type rapeseed. Since winter-type rapeseed lines start their germination late in the autumn and resume growth early in the spring, storage lipids with lower melting points may enable their growth at the relatively low temperatures and allow them to activate their metabolism early in the spring.

## 4. Conclusions

High-throughput molecular phenotyping techniques such as UPLC–MS allow highly sensitive profiling of many lipid molecules at the same time. This profiling may be very useful in oilseed crop breeding enabling the comparison of lipid content among different lines and varieties, as well as in the studies of plant biochemical pathways on the whole. The comparison of the TAG composition in sunflower and rapeseed samples revealed the similarity in the content of the most intense TAGs 54:3, 54:4, 54:5, and 54:6 between these two plants. Apart from those mentioned above, the current technique used also allowed to find significant differences in the TAG amounts between sunflower and rapeseed.

Importantly, winter rapeseed lines were demonstrated to be enriched in the TAGs with higher amount of double bonds (52:4, 52:5, 52:6, 54:6, and 54:7), while spring rapeseed lines were shown to contain mostly the TAGs with higher level of fatty acid chain saturation (48:1, 48:2, 54:2, and 54:3). The presence of TAGs with more than three double bonds has a significant impact on the plant’s resistance to cold. The observed differences in the saturation levels of TAGs in the winter-type and spring-type rapeseed may provide new insights into the cold tolerance mechanisms in plants which is highly important in terms of global climate change.

## Supplementary Materials

The following are available online at http://www.mdpi.com/2218-273X/9/1/9/s1, Figure S1: Mean TAG intensities in blank and plant samples, Table S1: Sunflower and rapeseed lines used in the present study, Table S2: Raw TAG intensities in plant and blank samples, Table S3: TAG mass fraction comparisons, Table S3A: Summary on the TAG mass fraction comparisons between rapeseed and sunflower, Table S3B: Summary on the TAG mass fraction comparisons between winter and summer rapeseed.
