# Supplementary figures and images for: UPLC–MS Triglyceride Profiling in Sunflower and Rapeseed Seeds
Source: Biomolecules. 2018 Dec 27;9(1):9. doi: 10.3390/biom9010009 (PMC6359410; doi:10.3390/biom9010009)

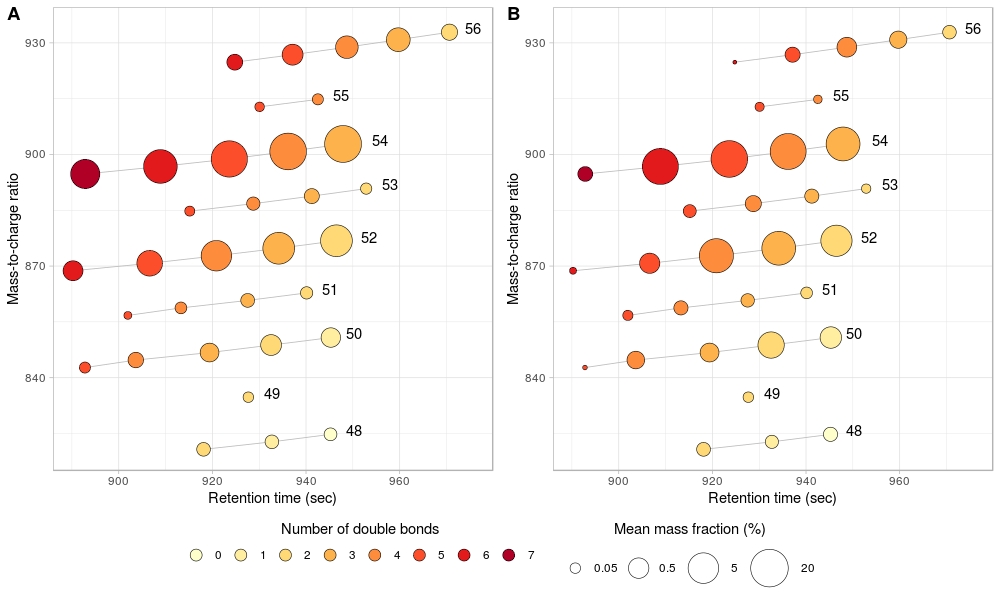

Supplement: Supplementary file 1 [file biomolecules-09-00009-s001.zip › Fig1.jpeg]
